# Detection rate and mutational landscape in extracranial arteriovenous malformations: a cohort study

**DOI:** 10.1186/s12916-026-04874-0

**Published:** 2026-04-16

**Authors:** Vanessa F. Schmidt, Denny Schanze, Richard Brill, Julius H. Loeser, Wibke Uller, Michael Doppler, Özlem Cangir, Susanne Hengst, Veronika Vielsmeier, Maciej Pech, Florian Obereisenbuchner, Mirjam Schirren, Alena Sint, Daniel Puhr-Westerheide, Sinan Deniz, Jakob B. W. Weiß, Beate Häberle, Alexandra Hartel, Alexandra Fröba-Pohl, Julia Haehl, Annegret Holm, Peter B. Sporns, Thomas Scherf, Jens Ricke, Silke Lassmann, Max Seidensticker, Walter A. Wohlgemuth, Melanie A. Kimm, Martin Zenker, Moritz Wildgruber, Friedrich G. Kapp

**Affiliations:** 1 Department of Radiology, LMU University Hospital, LMU Munich, Marchioninistr. 15, Munich, 81377 Germany; 2 Interdisciplinary Center for Vascular Anomalies (IZGA), LMU University Hospital, LMU Munich, Munich, Germany; 3 Institute of Human Genetics, University Hospital Magdeburg, Magdeburg, Germany; 4 Clinic and Policlinic of Radiology, Martin-Luther University Halle-Wittenberg, Halle (Saale), Germany; 5 Department of Diagnostic and Interventional Radiology, University of Freiburg Medical Centre, Medical Faculty of the University of Freiburg, Freiburg, Germany; 6 Department of Pediatric Surgery, St. Franziskus Hospital Münster, Münster, Germany; 7 Department of Radiology, Center for Vascular Malformations, Klinikum Barnim GmbH, Werner Forssmann Hospital, Eberswalde, Germany; 8 Department of Otorhinolaryngology, Regensburg University Medical Center, Regensburg, Germany; 9 Departments of Radiology and Nuclear Medicine, Otto-Von-Guericke University of Magdeburg, Magdeburg, Germany; 10 Department of Plastic and Hand Surgery, Medical Faculty, University of Freiburg Medical Center, University of Freiburg, Freiburg, Germany; 11 Department of Pediatric Surgery, Dr. Von Hauner Children’s Hospital, LMU University Hospital, LMU Munich, Munich, Germany; 12 Vascular Biology Program, Department of Surgery, Boston Children’s Hospital, Harvard Medical School, Boston, MA USA; 13 Department of Pediatric Hematology, Oncology and Stem Cell Transplantation, Children’s Hospital, Medical Center - University of Freiburg, Faculty of Medicine, University of Freiburg, Freiburg, Germany; 14 Department of Radiology and Neuroradiology, Stadtspital Zürich, Zurich, Switzerland; 15 Department of Neuroradiology, Clinic for Radiology and Nuclear Medicine, University of Basel and University Hospital Basel, Basel, Switzerland; 16 Institute for Surgical Pathology, Medical Center, Faculty of Medicine, University of Freiburg, University of Freiburg, Freiburg, Germany

**Keywords:** Arteriovenous malformations, AVM, Mosaicism, Pathogenic variants, Detection rate

## Abstract

**Background:**

This study aimed to assess the detection rate and spectrum of pathogenic variants (PVs) and candidate variants (variants of uncertain significance, VUS) in AVM patients.

**Methods:**

In this retrospective multicenter cohort study, tissue and blood samples were collected from 114 patients with extracranial AVMs during treatment or when clinically indicated, for dedicated molecular genetic analyses. PVs (solved) and VUS were detected by targeted sequencing on DNA using gene panels analyzing genes suspected to be associated with AVMs. Unsolved cases were further categorized into unrestricted and restricted, with the latter reflecting methodological limitations. Subgroup analyses were carried out based on affected genes to explore associated genotype–phenotype correlations.

**Results:**

PVs were identified in 80.7% (92/114) and VUS in 5.3% (6/114), resulting in a total detection rate of 86.0% (98/114). Unsolved cases accounted for 11.4% (13/114) including 6/13 (46.2%) with methodological restrictions. Somatic PVs were most frequent in KRAS (21.1%, 24/114), *MAP2K1* (17.5%, 20/114), *HRAS* (8.8%, 10/114), and *BRAF* (7.9%, 9/114). Germline variants were found in *RASA1* (7.0%, 8/114), *PTEN* (5.3%, 6/114), and *EPHB4* (3.5%, 4/114). In a few cases, somatic variants in *RASA1* (1.8%, 2/114) and *PTEN* (2.6%, 3/114) were identified. Additional PVs occurred in *PIK3CA* (3.5%, 4/114), *SOS1* (2.6%, 3/114), *GNAQ* (1.8%, 2/114), and *RIT1, RAF1*, and *GNA14* (each 0.9%, 1/114). Within the RAS/MAPK pathway, *RAS* variants (*KRAS*, *HRAS*) were linked to more severe clinical stages (65.6% vs. 40.0% *MAP2K1 and 37.5*% *BRAF*, *p* = 0.027) and higher relapse rates (55.6% vs. 43.8% *MAP2K1 and 0*% *BRAF*, *p* = 0.049). Germline variants showed a distinct distribution pattern with more syndromic presentations (61.1% vs. 15.6%, *p* < 0.001) compared to mosaic variants.

**Conclusions:**

Broad and sensitive testing enables a high detection rate of causative variants in AVMs. PVs and VUS detected reveal a broader genetic spectrum than previously recognized. Somatic *RAS* PVs were associated with more advanced disease stages and higher relapse rates than *MAP2K1* and *BRAF* variants, while germline variants were more frequently linked to syndromic patterns.

**Supplementary Information:**

The online version contains supplementary material available at 10.1186/s12916-026-04874-0.

## Background

Vascular malformations are congenital anomalies that are further classified into developmental anomalies of named vessels, slow-flow malformations (predominantly venous and lymphatic) as well as fast-flow arteriovenous malformations (AVMs). AVMs are associated with higher morbidity, risk of rapid progression, and challenging treatment [1–3]. Complete remission is often not achievable, and residual lesions typically continue to progress. The 2025 update of the ISSVA classification emphasized the integration of genetic findings, deepening insight into the pathogenesis of these rare lesions. Expanding knowledge into the genetic and molecular basis of vascular malformations have reshaped the understanding of their disease mechanisms, paving the way for improved patient stratification and individualized therapeutic approaches [4, 5]. Sporadic vascular malformations are frequently caused by somatic gain-of-function variants in genes encoding components of key signaling pathways that regulate cell growth and differentiation [6]. The broad spectrum of clinical phenotypes is thought to reflect the affected cell type, the timing of the mutational event, and the extent and nature of pathway activation. In conditions caused by mutations affecting the PI3K/AKT/mTOR signaling pathway, genotype-based stratification has led to early clinical trials of targeted therapies [7]. While vascular malformations were historically regarded as static dysplastic anomalies, increasing evidence of activated growth- and proliferation-related pathways suggests that these lesions possess proliferative potential [8]. Within this context, robust cohorts providing reliable data on detection rates, defining the variant spectrum, and clarifying genotype–phenotype associations are still lacking. Here, we present a multicenter study in which ultra-deep next-generation sequencing of DNA samples from affected tissue or blood identified pathogenic variants (PVs), report their detection rate, and analyze genotype–phenotype correlations with potential clinical implications.

## Methods

### Aim of the study

This study primarily aimed to report the diagnostic yield and mutational landscape following molecular genetic testing in a multicenter AVMs cohort, and secondarily to analyze genotype–phenotype correlations with potential clinical implications. Clinical features, lesion extension, and progression rates are included as part of the phenotype.

### Patient cohort and sample collection

This retrospective multicenter cohort study was conducted among interdisciplinary vascular anomalies centers of six university hospitals in Germany. Included are patients with a clinical diagnosis of AVM, who were selected for genetic testing between 2019 and 2025. All genetic analyses were performed based on clinical indications. In accordance with national regulations (German Genetic Diagnostics Act, GenDG), all participants provided informed consent for diagnostic testing as well as for the scientific use of the results, including radiological and clinical images. Furthermore, retrospective analyses were approved by the local ethics committee (University Hospital, LMU Munich, protocol no: 23–0337). The Strengthening the Reporting of Genetic Association Studies (STREGA) [9] guidelines were used for appropriate reporting, see Additional file 1: Table 1. In general, off-label therapy (e.g., trametinib or dabrafenib) was available for patients at all participating centers and could be reimbursed by the patients’ insurance providers after approval of individual cost-coverage applications, as no suitable clinical trials were recruiting.

Part of the cohort (22/114 patients) has already been published previously, see Additional file 2: Table 2 [5]. Core needle biopsies were performed under ultrasound guidance close to the AVM nidus. All patients with AVMs that underwent molecular genetic testing within the specified period were included, regardless of whether a PV was detected or not. Analysis was performed in corresponding fresh or formalin-fixed, paraffin-embedded (FFPE) tissue, or ethylenediaminetetraacetic acid (EDTA) blood. Exclusion criteria were clinical, pathological, or radiological doubts regarding the diagnosis of an AVM. Thus, detection rate of PVs (solved) and of candidate variants (variants of uncertain significance, VUS), as well as the rate of unsolved cases were calculated. Unsolved cases were further categorized into unrestricted and restricted genetic testing. In the latter, methodological limitations may explain the absence of detectable variants in the corresponding sample (e.g. availability of leukocyte DNA only, poor DNA quality of FFPE samples). Demographics, medical history, clinical, and radiological data as well as treatment course and follow-up assessments were collected. Clinical suspicion of disease recurrence was confirmed by MRI. Disease progression was defined as increase or reoccurrence of clinical symptoms after treatment as well as increased lesion size or newly perfused vessels compared to preceeding imaging findings.

Details about DNA extraction, variant screening, and genetic analysis are listed in the Additional file 3. The gene content also evolved over time (a complete list of genes/gene hotspots covered by the employed enrichment kit is available in Additional file 4: Table 3).

### Statistics

Data are presented as mean (± SD) or median (range, minimum–maximum). Subgroup analyses were performed related to the affected genes or to defined variant groups according to overarching features (e.g. germline versus mosaic variants). Cramers' V test and Fisher's exact test (if n < 5 in > 20% of cell counts) were applied for categorial data and small sample sizes to assess the association of the different PV carriers or group with clinical phenotypes such as lesion localization, lesion tissue involvement (one versus several tissue types), Cho and Schobinger classification, relapse (yes/no), and distribution pattern (isolated, multifocal, syndromic) according to 2025 ISSVA classification [10]. Multifocal lesions were defined as the presence of two or more anatomically distinct vascular malformations in the same patient (e.g., CM-AVM), without association with additional congenital anomalies or systemic features. Syndromic lesion was defined as an AVM occurring as part of a clinically recognizable syndrome, in association with other congenital anomalies or systemic manifestations (e.g., soft tissue or skeletal hypertrophy). Binary and multinominal logistic regression models were performed to examine potential confounders, including age, sex, and treatment methods. Analysis was conducted using SPSS (Version 26.0, Armonk, NY: IBM Corp.), all p-values reported are two-tailed.

## Results

### Patient characteristics and clinical presentation

All patients included (*n* = 114, 100%) presented with clinically and radiologically confirmed extracranial AVMs [10], and were managed interdisciplinarily according to clinical features, Schobinger stage [11], and patient preference (embolization, surgery/amputation, laser, targeted medical therapy, observation). The cohort consisted of 60/114 (52.6%) females and 54/114 (47.4%) males as well as 49/114 (43.0%) children (< 18 years, mean age of 8.9 years ± 4.7 years). Genetic testing was performed at a mean age of 22.3 years (± 14.7 years). AVMs were located on lower extremities in 43/114 patients (37.7%), followed by head and neck (33/114, 28.9%), upper extremities (14/114, 12.3%), trunk (7/114, 6.1%), and parenchymatous organs (2/114, 1.8%). The remaining lesions (15/114, 17.2%) extensively involved two or more anatomical regions (Table 1). Related to affected tissue types (cutaneous, subcutaneous, intramuscular, intraosseous, parenchymatous, nerve), 35/114 AVMs (30.7%) were limited to one type, 79/114 (69.3%) AVMs involved several types (Table 1). An extension of the AVM into parts of the central nervous system was observed in 6/114 patients (5.3%). The clinical stage of AVMs according to Schobinger [11] (*n* = 110) presented as follows: stage 1 (12/110, 10.9%), stage 2 (44/110, 40.0%), stage 3 (47/110, 42.7%), and stage 4 (7/110, 6.4%). AVMs were classified angiographically according to Cho [12] (*n* = 96): type I (2/96, 2.1%), type II (8/96, 8.3%), type IIIa (37/96, 38.5%), and type IIIb (49/96, 51.0%; Table 1).

**Table 1:** Patient characteristics and clinical presentation of study cohort

	Total cohort
*Age (years)*	
*Mean (*± *SD)*	22.3 (± 14.7)
*Sex*	
* Female*	60/114 (52.6%)
*Lesion localization*	
*Lower Extremity*	43/114 (37.7%)
*Head and neck*	33/114 (28.9%)
*Upper extremity*	14/114 (12.3%)
*Trunk*	7/114 (6.1%)
*Intraabdominal*	2/114 (1.8%)
*Extensive lesions (*> *1 anatomical regions)*	15/114 (13.2%)
Cho classification for AVMs^*^	
*I*	2/96 (2.1%)
*II*	8/96 (8.3%)
*IIIa*	37/96 (38.5%)
*IIIb*	49/96 (51.0%)
*Schobinger classification for AVMs*^**†**^	
1	12/110 (10.9%)
2	44/110 (40.0%)
3	47/110 (42.7%)
4	7/110 (6.4%)
*Affected tissue types*	
*Cutaneous*	55/114 (48.2%)
*Subcutaneous*	88/114 (77.2%)
*Intramuscular*	67/114 (58.8%)
*Osseous*	16/114 (14.0%)
*Parenchymatous*	2/114 (1.8%)
*AVM involving one tissue type*	35/114 (30.7%)
*AVM involving several tissue types*	79/114 (69.3%)
*Extension into central nervous system*	
*Yes*	6/114 (5.3%)
*No*	108/114 (94.7%)
*Distribution according to ISSVA*^+^ *classification 2025*	
*Isolated*	74/114 (64.9%)
*Multifocal*	13/114 (11.4%)
*Syndromic*	27/114 (23.7%)
*Accompanying hypertrophy*	
*Yes*	53/114 (46.5%)
*Soft tissue*	41/114 (36.0%)
*Bone*	1/114 (0.9%)
*Both*	11/114 (9.6%)
*No*	61/114 (53.5%)

^*^Cho classification according to Cho, Do [12] ^**†**^Schobinger classification according to Kohout, et al. [11]. ^+^ISSVA classification of vascular anomalies ©2025 international society for the study of vascular anomalies available at "issva.org/classification"

### Treatment course and follow-up

In 49/114 (43.0%) cases, the AVMs were treated by minimally invasive interventional radiology (embolotherapy), in 23/114 (20.2%) patients a combined approach of IR and surgical resection and in 11/114 (9.6%) of IR and targeted medical therapy. Six of 114 (5.3%) patients underwent surgery alone, 3/114 (2.6%) targeted medical therapies alone, and 12/114 (10.5%) patients did not undergo any treatment. The median number of invasive treatment sessions was 3 (0–41), the median follow-up time 22 months (7–36 months). Overall, 32 of 76 patients with follow-up data (42.1%) experienced a relapse in the clinical course after treatment. At the last follow-up, 14/83 (16.9%) lesions demonstrated complete regression, 50/83 (60.2%) partial regression, 14/83 (16.9%) AVMs remained stable, while 5/83 (6.0%) showed progression. In 43/114 patients (37.7%; based on the detected variants: 43/98, 43.9%), clinical management was modified according to the genetic findings, with subsequent initiation of targeted medical therapy in 12/114 cases (10.5%; 12/98, 12.2%), impact on family counseling in 18/114 cases (15.8%; 18/98, 18.4%), or even revision of the clinically suspected diagnosis in 13/114 cases (11.4%; 13/98, 13.3%). Treatment modalities and types of targeted medical therapy are detailed in Table 2.

**Table 2:** Treatment course and follow-up

	Total cohort
*Treatment*	
*None*	12/114 (10.5%)
*Yes*	102/114 (89.5%)
*IR*^+^ *only*	49/114 (43.0%)
*IR*^+^ *and surgery*	23/114 (20.2%)
*IR*^+^ *and targeted medical therapy*	11/114 (9.6%)
*Surgery only*	6/114 (5.3%)
*IR*^+^*, surgery, and targeted medical therapy*	6/114 (5.3%)
*Targeted medical therapy only*	3/114 (2.6%)
*IR*^+^ *and laser*	3/114 (2.6%)
*Laser only*	1/114 (0.9%)
*Type of targeted medical therapy*	20/114 (17.5%)
*Trametinib only*	6/114 (5.3%)
*Sirolimus only*	5/114 (4.4%)
*Thalidomide only*	3/114 (2.6%)
*Trametinib and Thalidomide*	2/114 (1.8%)
*Bevacizumab only*	1/114 (0.9%)
*Dabrafenib only*	1/114 (0.9%)
*Bevacizumab and Trametinib*	1/114 (0.9%)
*Lenalidomide and Trametinib*	1/114 (0.9%)
*Number of invasive treatment session, median (range)*	3 (0–41)
*Treatment response at last follow-up*	
*Complete regression*	14/83 (16.9%)^*^
*Partial regression*	50/83 (60.2%)
*Stable*	14/83 (16.9%)
*Progression*	5/83 (6.0%)
*Relapse during treatment course*	
*Yes*	32/76 (42.1%)
*Management adapted by genetic findings*	43/114 (37.7%)
*Targeted medical therapy*	12/114 (10.5%)
*Impact on family counseling*	18/114 (15.8%)
*Revision of the clinically suspected diagnosis*	13/114 (11.4%)

^+^*IR* Interventional radiology, e.g. embolization. ^*^Including three cases of surgical amputation

### Detection rate and spectrum of PVs

**Fig. 1:**
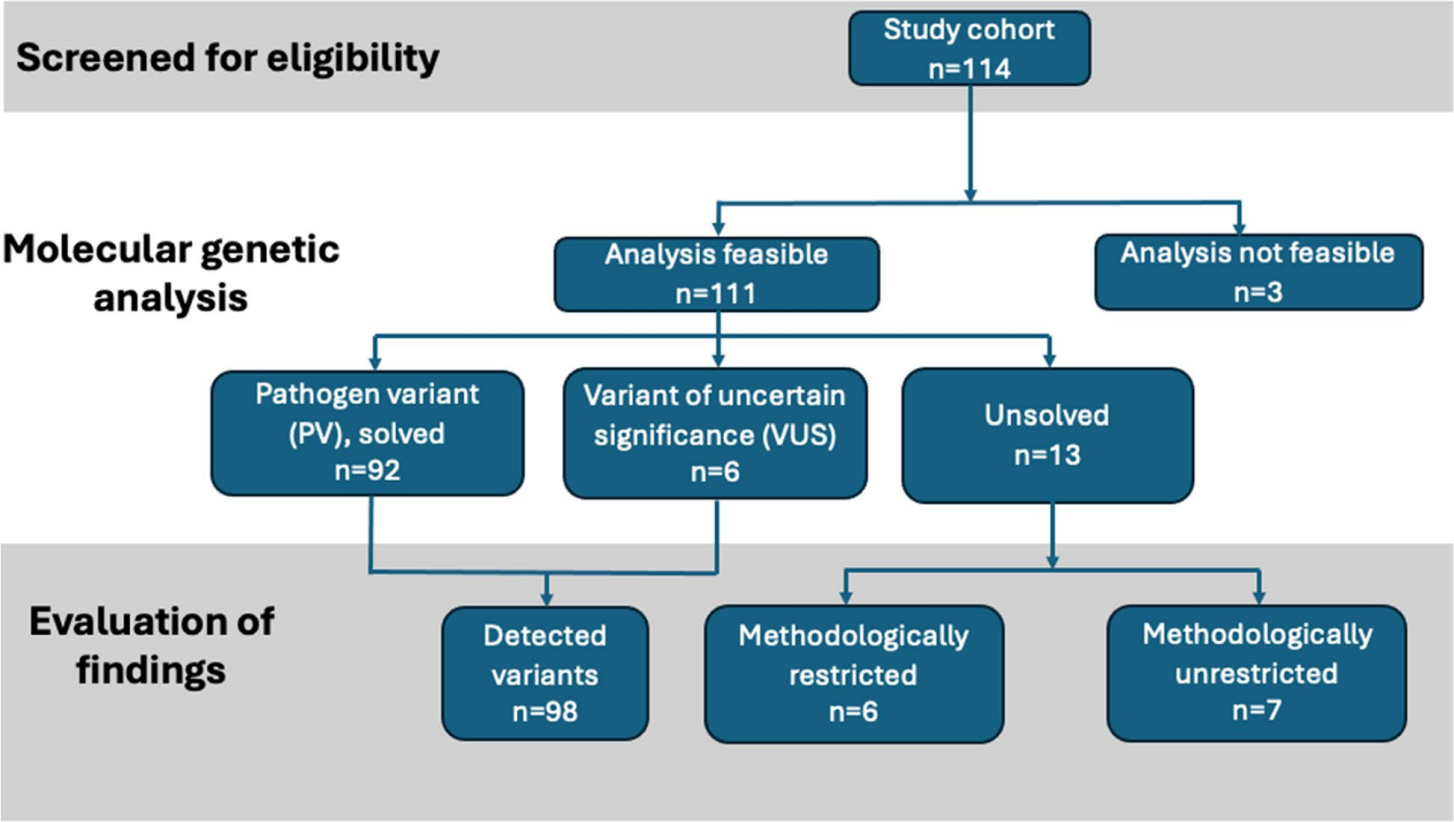
Consort flow chart presenting AVM cohort size and variant detection rate

PVs (pathogenic or likely pathogenic variants) were found in 80.7% (92/114) and candidate variants (variants of uncertain significance, VUS) in 5.3% (6/114), resulting in a total detection rate of 86.0% (98/114, Fig. 1). Thirteen of 114 cases (11.4%) remained unsolved, further divided into methodological unrestricted in 7/13 (53.8%; 6.0% of total cohort) and restricted in 6/13 (46.2%; 5.3% of total cohort) cases. The detailed methodological limitations that may explain the absence of detectable variants in the latter group can be found in Table 3. In three of 114 (2.6%) cases the genetic analysis was not feasible due to insufficient DNA quality from FFPE samples (Table 3).

**Table 3:** Molecular genetic analysis and detection rate of PVs

	Total cohort
*Molecular genetic analysis*	114/114 (100%)
*Testing not feasible*	3/114 (2.6%)
FFPE without interpretable result^*^	1/114 (0.9%)
Sanger sequencing on FFPE DNA only^*^	1/114 (0.9%)
*FFPE with 166* × *coverage, repeated analysis with 184* × *coverage only*	1/114 (0.9%)
*Testing feasible*	111/114 (97.4%)
*Total detection rate*	98/114 (86.0%)
*Pathogenic variants (PVs), solved*	92/114 (80.7%)
*Variants of uncertain significance (VUS)*	6/114 (5.3%)
*Unsolved cases*	13/114 (11.4%)
*Methodological unrestricted*	7/114 (6.1%)
*Methodological restricted*	6/114 (5.3%)
*cfDNA only*	2/114 (1.8%)
*Germline testing only (EDTA-blood)*	2/114 (1.8%)
*Limited gene panel*	1/114 (0.9%)

*CfDN* circulating-free deoxyribonucleic acid, *EDTA* ethylenediaminetetraacetic acid, *FFPE* Formalin-fixed, paraffin-embedded. ^*^No adequate material for repeated analyses

**Fig. 2:**
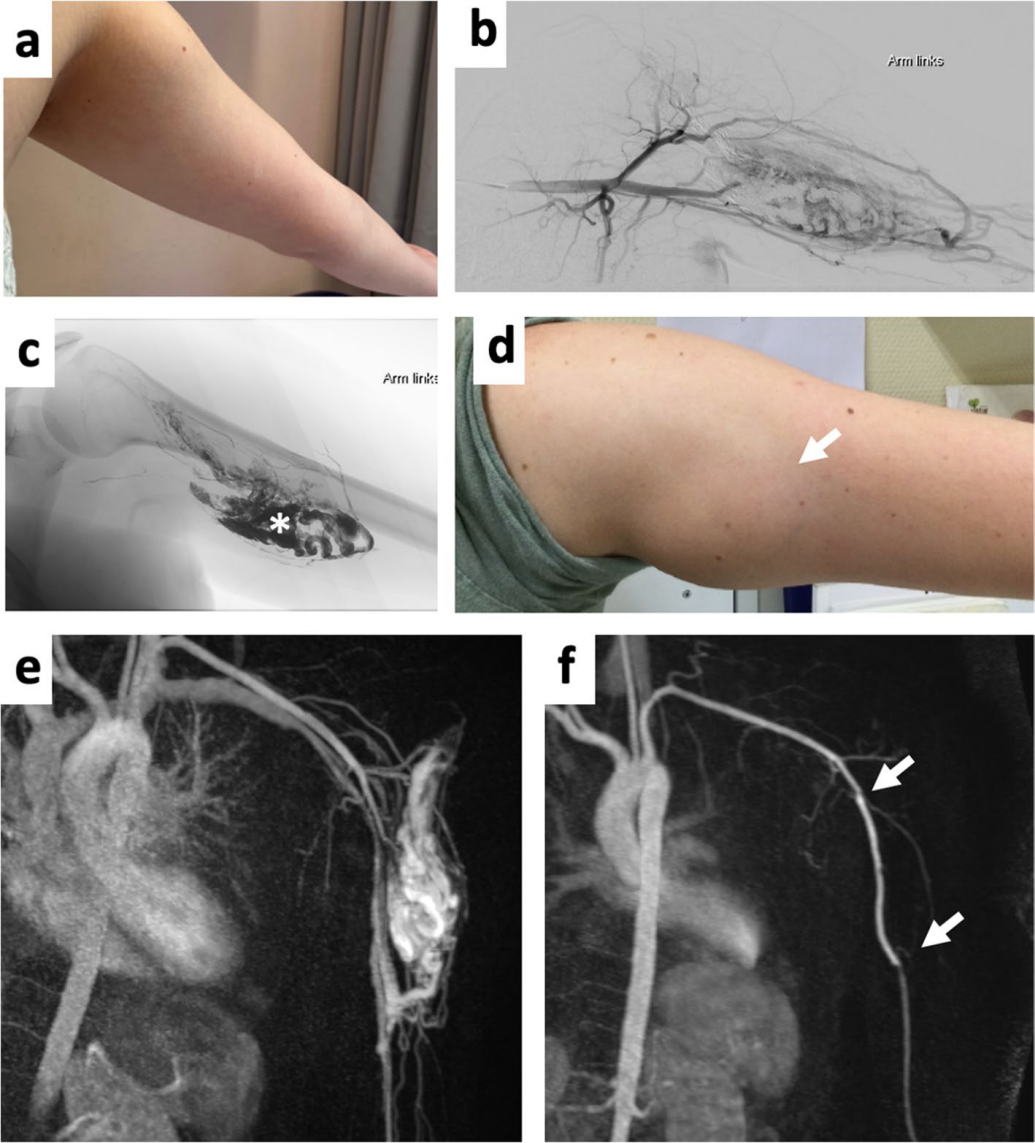
(Pat. No. 8). 27-year-old female patient with an arteriovenous malformation (AVM) of the left upper arm and a KRAS pathogenic variant. **a** Clinical presentation of the AVM after two prior embolization procedures (**a**), **b**, **c** Digital subtraction angiography (DSA) images presenting the angioarchitecture of the lesion and the remaining liquid embolization material (asterisk). **d** Clinical presentation of the AVM at clinical relapse with a clearly visible increase in size compared to (**a**), observed after an interval of 5 months (**b**, arrow). **e.** Time-resolved angiography with interleaved stochastic trajectories angiography (TWIST) magnetic resonance angiography (MRA) image showing the marked hyperperfusion and newly vascularized components of the progressive AVM. **f** TWIST MRA image after surgical resection of the AVM, including removal of the long head of the biceps and vascular and nerve dissection. Due to involvement of the brachial artery, a venous interposition graft from the right thigh was performed to restore and maintain blood flow to the arm (arrows)

**Fig. 3:**
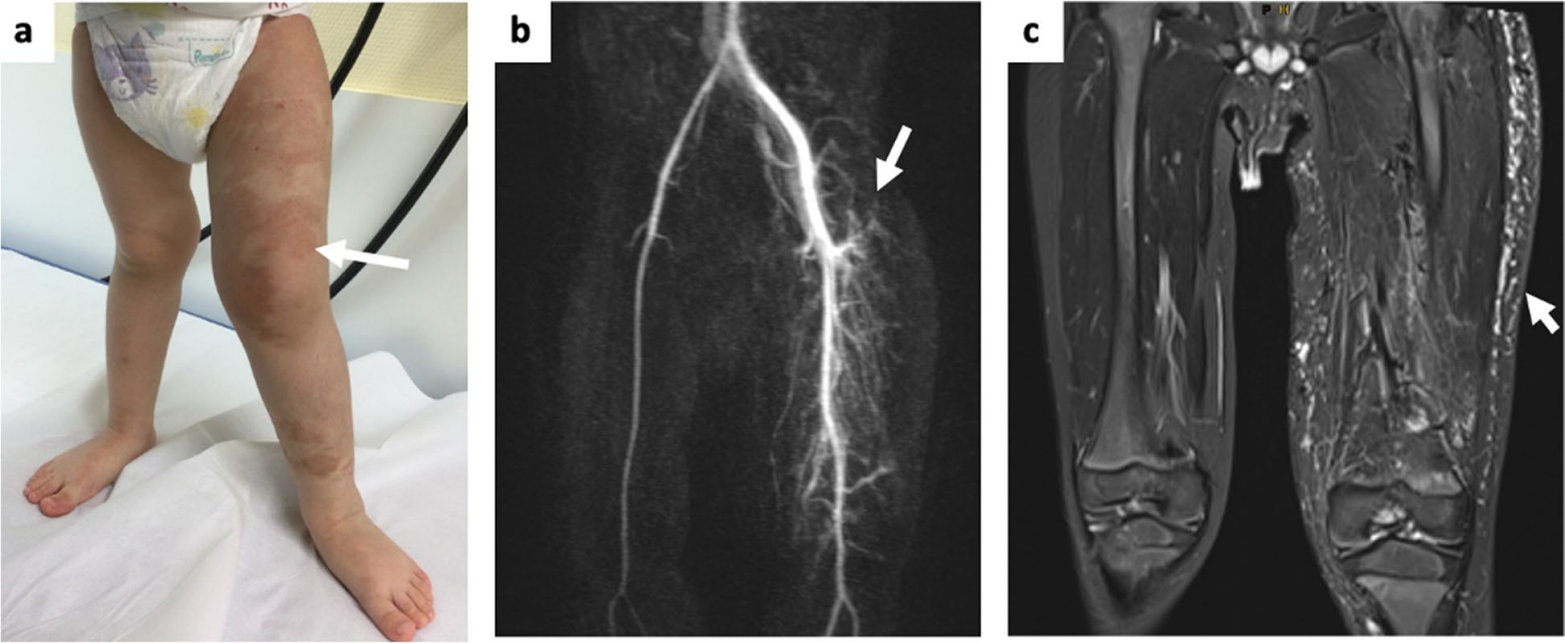
(Pat. No. 57). 2-year-old male patient presenting with a Parkes Weber syndrome-like phenotype of the left leg and somatic *BRAF* pathogenic variant. **a** Clinical presentation of the affected left leg with soft tissue hypertrophy of the thigh and confluent capillary malformations (arrow) with noticeable hyperthermia; furthermore, leg lengths discrepancy (left > right, 2 cm) was observed. **b** Time-resolved angiography with interleaved stochastic trajectories (TWIST) magnetic resonance angiography (MRA) image showing fine-fistulous AVM-components of the left thigh (arrow). **c.** T2-weighted turbo spin echo (TSE) MR image presenting the extent of the AVM components and accompanied soft tissue hypertrophy and edema (arrow)

**Fig. 4:**
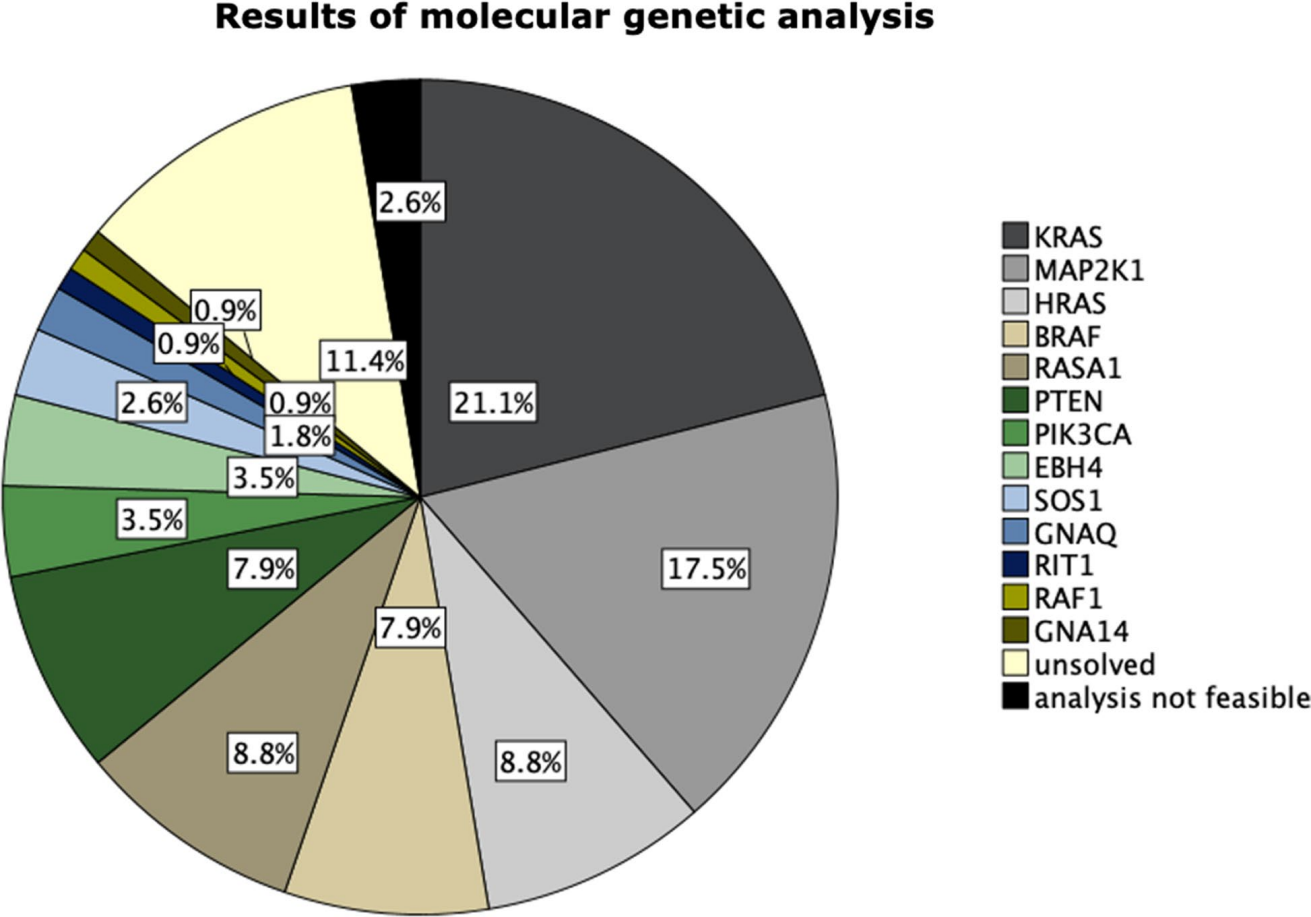
Results of molecular genetic analysis and variant spectrum in AVM cohort

The identified PVs involved the genes *KRAS* in 24/114 (21.1%, Fig. 2), *MAP2K1* in 20/114 (17.5%), *HRAS* in 10/114 (8.8%), and *BRAF* in 9/114 (7.9%, Fig. 3) patients. Eight of 114 (7.0%) patients carried a germline *RASA1* variant, while two (2/114, 1.8%) patients harbored a somatic pathogenic *RASA1* variant. Six of 114 (5.3%) patients were found to have a germline *PTEN* variant and three 3/114 (2.6%) patients a somatic pathogenic *PTEN* variant. Germline *EBH4* variants were observed in 4/114 (3.5%), identically in number to *PIK3CA* somatic variants (4/114, 3.5%). A somatic PV in *SOS1* was detected in 3/114 (2.6%) cases, followed by two *GNAQ* PVs (2/114, 1.8%) as well as one *RIT1* PV, one *RAF1* VUS*,* and one *GNA14* PV (each 1/114, 0.9%, Fig. 4). Variant allele frequencies (VAFs) in DNA from lesional tissue samples ranged from 0.3% to 23.0% (Additional file 2: Table 2) with VAFs < 1.0% in 3/114 cases (2.6%) and VAFs < 3.0% in 12/114 cases (10.5%).

Together gene variants in *KRAS, HRAS, BRAF,* and *MAP2K1* accounted for 55.3% of cases overall (63/114) and 78.8% of cases with somatic variants (63/80). While *KRAS, BRAF,* and *MAP2K1* variants predominantly involved the mutational hotspots for oncogenic variants at codons 12 and 61 (*KRAS*), 600 (*BRAF*), and 56/57 (*MAP2K1), HRAS* PVs were exclusively indels affecting the switch 2 region of the protein. This observation is in line with previous reports [13, 14].

### Genotype–phenotype associations

When comparing between cases with PVs in the *RAS, MAP2K1*, and *BRAF* genes, the *RAS* group showed a more severe clinical stage according to Schobinger (stage 2 in *RAS*: 65.6% vs. 40.0% and 37.5% in *MAP2K1* and *BRAF*, *p* = 0.027) and a higher relapse rate (55.6% in RAS vs. 43.8% and 0.0% in *MAP2K1* and *BRAF*, *p* = 0.049). Potential confounders did not show a significant effect on Schobinger stage (multinominal regression; age: OR = 1.03; 95% CI: 0.93–1.14; *p* = 0.599; sex: OR = 2.32; 95% CI: 0.16–34.03; *p* = 0.540) or on the occurrence of relapse (binary regression modell; age: OR = 1.01; 95% CI: 0.96–1.05; *p* = 0.820; sex: OR = 1.43; 95% CI: 0.40–5.09; *p* = 0.586; treatment method: OR = 1.08; 95% CI: 0.78–1.49; *p* = 0.658). Regarding the tissue involvement, AVMs with *BRAF* variants involved a single tissue type in 44.4% compared to *RAS* in 32.4% and *MAP2K1* in 35.0%, though this was not statistically significant. No remarkable differences were observed in the prevalence of clinical manifestations, such as anatomical lesion localization, accompanied hypertrophy, or in the angiographic classification according to Cho (all *p* > 0.05, Table 4).

**Table 4:** Comparison of clinical characteristics between PVs of RAS/MAPK-pathway

Clinical characteristics	RAS	BRAF	MAP2K1	p-value
*Lesion localization*				*p* = 0.301^#^
*Lower Extremity*	15/34 (44.1%)	4/9 (44.4%)	5/20 (25.0%)	
*Head and neck*	8/34 (23.5%)	2/9 (22.2%)	9/20 (45.0%)	
*Upper extremity*	5/34 (14.7%)	0/9 (0.0%)	3/20 (15.0%)	
*Trunk*	4/34 (11.8%)	1/9 (11.1%)	1/20 (5.0%)	
*Extensive lesions (two or more anatomical regions)*	2/34 (5.9%)	2/9 (22.2%)	2/20 (10.0%)	
Cho classification for AVMs^*^				*p* = 0.817^#^
*I*	1/31 (3.2%)	0/7 (0.0%)	0/17 (0.0%)	
*II*	3/31 (9.7%)	0/7 (0.0%)	0/17 (0.0%)	
*IIIa*	13/31 (41.9%)	4/7 (57.1%)	7/17 (41.2%)	
*IIIb*	14/31 (45.2%)	3/7 (42.9%)	10/17 (58.8%)	
*Schobinger classification for AVMs*^**†**^				**p = 0.027**^**#**^
1	0/32 (0.0%)	3/8 (37.5%)	2/20 (10.0%)	
2	10/32 (31.2%)	2/8 (25.0%)	8/20 (40.0%)	
3	21/32 (65.6%)	3/8 (37.5%)	8/20 (40.0%)	
4	1/32 (3.1%)	0/8 (0.0%)	2/20 (10.0%)	
*Involvement of tissue types*				*p* = 0.795^‡^
*One tissue type*	11/34 (32.4%)	4/9 (44.4%)	7/20 (35.0%)	
*Several tissue types*	23/34 (67.6%)	5/9 (55.6%)	13/20 (65.0%)	
*Distribution according to ISSVA 2025 classification*^+^				*p* < 0.341^#^
*Isolated*	23/34 (67.6%)	7/9 (77.8%)	18/20 (90.0%)	
*Multifocal*	1/34 (29.4%)	1/9 (1.1%)	1/20 (5.0%)	
*Syndromic*	8/34 (23.5%)	1/9 (1.1%)	1/20 (5.0%)	
*Accompanied hypertrophy*				*p* = 0.641^‡^
*yes*	16/34 (47.1%)	3/9 (33.3%)	7/20 (35.0%)	
*no*	18/34 (52.9%)	6/9 (66.7%)	13/20 (65.0%)	
*Relapse during medical/invasive treatment*				**p = 0.049**^**‡**^
*Yes*	10/18 (55.6%)	0/6 (0.0%)	7/16 (43.8%)	
*No*	8/18 (44.4%)	6/6 (100.0%)	9/16 (56.3%)	

^*^Cho classification according to Cho, Do [12]**; **^**†**^Schobinger classification according to Kohout et al. [11]. ^‡^Chi-square test. ^#^Fisher's exact test if n < 5 in > 20% of cell counts. ^+^ISSVA classification of vascular anomalies ©2025 international society for the study of vascular anomalies available at "issva.org/classification"

When comparing germline and mosaic variants, we found no relevant association with clinical stages according to Schobinger (*p* = 0.355), angiographic classification according to Cho (*p* = 0.950), or recurrence rate (*p* = 0.940). As expected, the assessed distribution pattern showed significant differences between the two groups, with more syndromic patterns in germline variants (61.1% vs. 15.6%, *p* < 0.001). AVMs due to germline mutations trended to involve more frequently two or more anatomical regions, although not statistically significant (27.8% vs. 10.4%, *p* = 0.08; Table 5).

**Table 5:** Comparison of clinical characteristics between germline and mosaic variants

Clinical characteristics	Germline variants	Mosaic variants	p-value
*Lesion localization*			*p* = 0.080^#^
*Lower Extremity*	8/18 (44.4%)	35/96 (36.5%)	
*Head and neck*	3/18 (16.7%)	30/96 (31.3%)	
*Upper extremity*	1/18 (5.6%)	13/96 (13.5%)	
*Trunk*	0/18 (0.0%)	7/96 (7.3%)	
*Parenchymatous organs*	1/18 (5.6%)	1/96 (5.6%)	
*Extensive lesions (two or more anatomical regions)*	5/18 (27.8%)	10/96 (10.4%)	
Cho classification for AVMs^*^			p = 0.950^#^
*I*	0/12 (0.0%)	2/84 (2.4%)	
*II*	1/12 (8.3%)	7/84 (8.3%)	
*IIIa*	4/12 (33.3%)	33/84 (39.3%)	
*IIIb*	7/12 (58.3%)	42/84 (50.0%)	
*Schobinger classification for AVMs*^**†**^			*p* = 0.355^#^
1	3/18 (16.7%)	9/92 (9.8%)	
2	8/18 (44.4%)	36/92 (39.1%)	
3	5/18 (27.8%)	42/92 (45.7%)	
4	2/18 (11.1%)	5/92 (5.4%)	
*Involvement of tissue types*			*p* = 0.395^‡^
*One tissue type*	4/18 (22.2%)	31/96 (32.3%)	
*Several tissue types*	14/18 (77.8%)	65/96 (67.7%)	
*Distribution according to ISSVA 2025 classification*^+^			**p < 0.001**^**#**^
*Isolated*	3/18 (16.7%)	71/96 (73.9%)	
*Multifocal*	4/18 (22.2%)	9/96 (9.4%)	
*Syndromic*	11/18 (61.1%)	16/96 (16.7%)	
*Accompanied hypertrophy*			*p* = 0.205^#^
*yes*	11/18 (61.1%)	42/96 (43.8%)	
*no*	7/18 (38.9%)	54/96 (56.3%)	
*Relapse during medical/invasive treatment*			p = 0.940^‡^
*Yes*	7/12 (58.3%)	32/56 (57.1%)	
*No*	5/12 (41.7%)	24/56 (42.9%)	

^*^Cho classification according to Cho, Do [12]**; **^**†**^Schobinger classification according to Kohout et al. [11]. ^‡^Chi-square test. ^#^Fisher's exact test if n < 5 in > 20% of cell counts. ^+^ISSVA classification of vascular anomalies ©2025 international society for the study of vascular anomalies available at "issva.org/classification"

No differences were observed between the detected variants and the unsolved cases when comparing clinical characteristics (all *p* > 0.05, Table 6).

**Table 6:** Comparison of clinical characteristics between unsolved cases and detected variants

Clinical characteristics	Unsolved cases	Detected variants^##^	p-value
*Lesion localization*			p = 0.140^#^
*Lower Extremity*	3/13 (23.1%)	36/92 (39.1%)	
*Head and neck*	3/13 (23.1%)	26/92 (28.3%)	
*Upper extremity*	4/13 (30.8%)	9/92 (9.8%)	
*Trunk*	0/13 (0.0%)	7/92 (7.6%)	
*Parenchymatous organs*	1/13 (7.7%)	1/92 (1.1%)	
*Extensive lesions (two or more anatomical regions)*	2/13 (15.4%)	13/92 (14.1%)	
Cho classification for AVMs^*^			p = 0.166^#^
*I*	1/11 (9.1%)	1/76 (1.3%)	
*II*	1/11 (9.1%)	4/76 (5.3%)	
*IIIa*	3/11 (27.3%)	31/76 (40.8%)	
*IIIb*	6/11 (54.5%)	40/76 (52.6%)	
*Schobinger classification for AVMs*^**†**^			p = 0.889^#^
1	2/12 (16.7%)	10/89 (11.2%)	
2	4/12 (33.3%)	34/89 (38.2%)	
3	5/12 (41.7%)	39/89 (43.8%)	
4	1/12 (8.3%)	6/89 (6.7%)	
*Involvement of tissue types*			p = 1.000^‡^
*One tissue type*	4/13 (30.8%)	29/92 (46.8%)	
*Several tissue types*	9/13 (69.2%)	63/92 (68.5%)	
*Distribution according to ISSVA 2025 classification*^+^			p = 0.740^#^
*Isolated*	9/13 (69.2%)	58/92 (63.0%)	
*Multifocal*	2/13 (15.4%)	10/92 (10.9%)	
*Syndromic*	2/13 (15.4%)	24/92 (26.1%)	
*Accompanied hypertrophy*			p = 1.000^‡^
*yes*	6/13 (46.2%)	42/92 (45.7%)	
*no*	7/13 (53.8%)	50/92 (54.3%)	
*Relapse during medical/invasive treatment*			p = 0.714^#^
*Yes*	4/8 (50.0%)	34/58 (58.6%)	
*No*	4/8 (50.0%)	24/58 (41.4%)	

^*^Cho classification according to Cho, Do [12]**; **^**†**^Schobinger classification according to Kohout et al. [11]. ^‡^Chi-square test. ^#^Fisher's exact test if n < 5 in > 20% of cell counts. ^+^ISSVA classification of vascular anomalies ©2025 international society for the study of vascular anomalies available at "issva.org/classification". ^##^Detected variants include both pathogenic variants (PVs, solved cases) and variants of unknown significance (VUS)

## Discussion

This retrospective multicenter study examined the detection rate and mutational landscape of PVs in AVMs and explored correlations between clinical characteristics and the implicated genes and variants. The diagnostic yield was high with variants detected in 86% of the cohort (80.7% PVs, 5.3% VUS). Most detected variants clustered within the RAS/MAPK signaling cascade, although the overall spectrum was broad containing 13 genes, both mosaic and germline variants, including not yet described variants. Syndromic distribution patterns were more frequent in germline variants than in mosaic variants, whereas no differences were observed for other clinical characteristics. Lesions with *RAS* PVs showed more advanced disease stages and higher relapse rates compared to *MAP2K1* and *BRAF* PVs in line with other publications.

This study's genetic detection rate of 86.0%, encompassing both PVs and VUS, is higher than reported in the AVM literature. This was further supported by the fact that only 6% of AVM cases remained unrestricted–unsolved, despite having undergone the maximum available genetic work-up with the current methodological possibilities. In 2018, Al-Olabi et al. identified somatic mosaic activating variants in the RAS/MAPK pathway (KRAS, BRAF, MAP2K1) in 6 of 9 patients (67%) using targeted NGS, thus they were among the first to establish these mutations as major drivers of sporadic AVMs [15]. A study by Green et al. identified somatic variants in *KRAS* and *MAP2K1* in 36% of 11 individuals with AVMs thereby using a broad approach with multiple methods including droplet digital PCR, Sanger sequencing, and high-depth exome sequencing [16]. El Sissy et al. identified PVs in 23.6% of blood samples drawn from 55 patients with AVMs, predominantly in *MAP2K1* and *KRAS* genes, with higher sensitivity near the AVM nidus [17]; this lower detection rate may be attributed to relevant methodological differences, as liquid biopsies applied cfDNA analysis, with lower sensitivity compared to tissue biopsies. Hernandez et al. reported 37 out of 54 patients (68.5%) with identified PVs, while 3.7% had VUS and 27.8% had negative results [18]. Their reported variant spectrum was predominantly composed of *MAP2K1* (12 cases), *KRAS* (8 cases), and *TEK* (7 cases), with some cases also harboring germline variants in *PTEN* and *RASA1 * [18]. In comparison, no *TEK* PVs were detected in any AVM of this cohort, but *TEK* is generally known to be involved in sporadic venous malformations [19]. A recent systematic review on genetic mutations included 69 studies across all type of vascular malformations reporting a causative variant in 40% of cases [20]. Overall, the high diagnostic yield in our study is likely attributable to the systematic use of high-sensitivity ultra-deep sequencing, with continuously evolving panels for somatic, germline, and combined analysis. Furthermore, enhanced quality of the tissue samples in our cohort may be attributable to the fact that the biopsies were deliberately taken as centrally as possible within the AVMs. In addition, the detection rate may have been influenced by cohort characteristics; for example, multifocal capillary malformation–AVM (CM-AVM) cases show a higher rate of RASA1 variants, with up to 70% of cases carrying a detectable mutation [21].

In this AVM cohort, mosaic or germline variants were identified in 13 different genes, representing a broad spectrum that also included some genes rarely reported in the context of AVMs, as well as novel genes. Compared to existing literature, we similarly observed a predominance of genes from the RAS/MAPK signaling pathway, primarily driven by *KRAS* and *MAP2K1*; however, we also noted a relevant proportion of *HRAS* (8.8%) and *BRAF* (7.9%) cases. In contrast, in the meta-analysis of Stor et al., only six and seven of 1,686 patients carried an activating somatic *HRAS* and *BRAF* variant, respectively. Variant calling of larger indels in *HRAS* cases may have been less efficient in preceding publications, potentially leading to missed variants. A similar rate of *BRAF* PVs of 8.7% was reported in the study by El Sissy et al. analyzing 23 AVM tissue samples [22]. Since *BRAF* cases have been generally described less frequently, less is known about this phenotype [23]. Our data indicate that these AVMs presented with lower disease stages and, in our cohort, showed no relapses in any of the affected patients (0%), despite also exhibiting complex angiographic phenotypes according to the Cho classification. In contrast, AVMs with PVs in *RAS* genes showed a higher relapse rate (55.6%) and more advanced disease stages. This pattern, representing a phenotype of primarily extensive and progressive AVMs, has also been previously reported by Stor et al. and Schmidt et al. [5, 20]. Overall, these data suggest that AVMs harboring a somatic RAS variant are associated with more advanced disease stages and higher relapse rates, indicating a need for further studies to determine optimal management strategies.

Yet, when interpreting the results it needs to be considered that patients in this cohort received various treatments, from minimal-invasive, open surgery to targeted pharmacological treatments. Statistically the treatment method itself did not represent a confounding factor for disease relapse. However, it has been shown that the treatment modality can potentially influence both the occurrence and the time to onset of relapse, for example embolization alone was reported to being associated with earlier recurrence compared to embolization plus resection [24, 25]. With respect to both disease stage and recurrence rate, AVMs with *MAP2K1* variants fell between *BRAF* and *RAS*, showing advanced stages and a relevant relapse rate (43.8%), but to a lesser extent than *RAS*. While AVMs with *RAS* variants commonly exhibited syndromic distribution patterns, *MAP2K1* AVMs were mostly found as isolated lesions, in line with prior reports [22].

PVs/VUS in *RASA1* and *PTEN* were predominantly found as germline variants in this cohort (8/10 and 6/9, respectively). The 8 germline cases and one of the two somatic *RASA1* cases presented with the typical phenotype of CM-AVM syndrome, with extensive, multifocal capillary malformations and associated AVMs, comparable to most reports of *RASA1* cases in literature [21, 26]. However, the remaining somatic *RASA1* case presented with an isolated AVM located in the palate. AVMs with *PTEN* variants were found in the lower extremities in 7 of 9 cases, and both germline and somatic *PTEN* variants exhibited intramuscular AVM components and surrounding hamartomatous changes. As part of the PTEN-hamartoma syndrome, these patients may not only present with vascular malformations but also with additional associated anomalies such as macrocephaly and multiple intracranial DVAs [27]. In addition to the germline variants in *RASA1* and *PTEN*, there were four germline variants in the *EPHB4* gene. Regarding the *EPHB4* cases, three were associated with capillary malformations, consistent with previous reports [28], whereas one case presented as an isolated parenchymal intra-abdominal AVM. As expected, comparison between germline and somatic variants revealed differences in distribution patterns of vascular lesions: germline variants were predominantly associated with syndromic patterns and tended to involve more tissue layers. In line with this, Valdivielso-Ramos et al. [29] reported that germline RASA1‐positive patients were more likely than RASA1-negative patients to present with a higher total number of clinical lesions, while Chen et al. demonstrated that germline pathogenic RASA1 variants were associated with a higher risk of multifocal CM compared to somatic variants [30]. No differences were observed in other clinical parameters, including angiographic findings, clinical classification, or relapse rates. These findings suggest that clinical presentation alone does not allow for a reliable distinction between germline and somatic mutations. Further, there is currently no evidence-based indication that germline PVs should be treated more intensively or proactively than somatic PVs.

*PIK3CA* variants were detectable in a small subset of this cohort including one VUS. The low percentage of *PIK3CA* variants in this study is consistent with previously described genetic spectra in AVMs. In general, *PIK3CA* is more associated with slow-flow vascular malformations and PIK3CA-realted overgrowth spectrum diseases, and clinical presentations are highly variable, ranging from solitary lesions to syndromic forms with hypertrophy and non-vascular anomalies [20]. Two *GNAQ* PVs (p.Arg183Gln) were found in this study, one of which presented with a Sturge-Weber syndrome like phenotype fitting with prior reports [31], but with an unexpectedly fast flow in the lesion. The remaining *GNAQ* case in our cohort presented with extensive infiltrative AVM of the head and neck, notably due to a *GNAQ* p.Gln209His mutation, which is a more aggressive GNAQ mutation often found in uveal melanoma. The one AVM patient with a confirmed *GNA14* PV presented with a head and neck AVM with components also involving the central nervous system. This variant has been only reported in a single case that harbored both a *PIK3CA* and a *GNA14* PV [32]. PVs in *SOS1* and a VUS in *RAF1* were identified in three and one patients, respectively. These genes have not yet been directly associated with AVMs, but are reported to be present in 3–20% in Noonan syndrome [33, 34]. *SOS1* activates RAS proteins while RAF1 functions downstream of RAS and upstream of MEK1/2, and thus both represent components of the canonical RAS/MAPK signaling pathway. In one AVM, a *RIT1* PV was found, this case was previously published and RIT1 was functionally characterized as novel gene implicated in the pathogenesis of AVMs [35]. As PVs in *SOS1, RAF1,* and *RIT1* genes present the capacity to hyperactivate the RAS-MAPK pathway, MEK inhibitors such as trametinib appear as medical targeted therapy option, which have already been used experimentally in individual cases [35]. In this cohort, one patient with a *SOS1* PV and the patient with a *RIT1* PV were treated successfully with trametinib. Overall, targeted medical therapy was initiated in 12.2% of the cases with detected variants following the genetic diagnosis. Patients treated with targeted therapy represent a relevant part of the 43 patients in this cohort whose clinical management was directly influenced by the genetic findings. Other patients were directed for family counseling or their diagnosis was revised due to the genetic finding. Similar observations including genetics-driven targeted therapy in 43 of 69 patients with vascular anomalies were reported by Li et al. [14].

Since no relevant differences in clinical characteristics were observed between AVMs with detected variants and those that remained unsolved, there currently is no rationale for pursuing a different therapeutic approach in AVMs without any identifiable underlying mutation. It can be anticipated that the proportion of unsolved cases will continue to decline, due to increasingly sensitive methods that allow the detection of very low VAFs in tissue samples, due to the identification of additional causative genes and due to further refinement of optimal material acquisition for genetic testing.

The limitations of this study include its retrospective design and the multicenter setting which may restrict available data (e.g. exact time point of relapse) as well as introduce inter-center heterogeneity in patient selection, diagnostic work-up, and treatment strategies, potentially affecting data consistency. Comparisons between subgroups were limited by the small and variable group sizes across individual PVs and VUS. Despite these limitations, this AVM study, retrieved robust molecular genetic findings in a real-world setting with substantial clinical implications, which may be further validated and expanded in future prospective studies.

## Conclusions

In this study, PVs and VUS have been detected in the majority of cases, predominantely within the RAS/MAPK pathway. Somatic *RAS* PVs correlated with advanced disease stages and higher relapse rates compared to *MAP2K1* and *BRAF* PVs, while germline variants were more often associated with syndromic patterns. Rare somatic variants in genes such as *SOS1, RAF1,* and *RIT1* were also identified, highlighting the expanding spectrum of contributors to AVM pathogenesis and allowing targeted medical therapy.

## Supplementary Information

Additional file 1. Table 1 Strengthening the Reporting of Genetic Association Studies (STREGA) guidelines.

Additional file 2. Table 2 Mutational spectrum and genotypic characterization of study cohort.

Additional file 3. Details about DNA extraction, variant screening, and genetic analysis.

Additional file 4. Table 3 Custom panel gene content.

## Data Availability

Detailed mutational spectra and genotypic characterization of the study cohort are provided in Additional file 2: Table 2. Due to privacy restrictions protecting study participants, the clinical datasets analyzed during the current study are not publicly available but may be obtained from the corresponding author upon reasonable request (vanessa.schmidt@med.uni-muenchen.de). Requests will generally be processed within 4 weeks.

## Abbreviations

AKT: Protein kinase B
AVM: Arteriovenous malformation
BRAF: V-Raf murine sarcoma viral oncogene homolog B
cfDNA: Circulating cell-free deoxyribonucleic acid
CM: Capillary malformation
EBHB4: Ephrin-receptor B4
EDTA: Ethylenediaminetetraacetic acid
FFPE: Formalin-fixed, paraffin-embedded
GAP: GTPase-activating protein
GNA14: G protein subunit alpha 14
GNAQ: Guanine nucleotide-binding protein G(q) subunit alpha
GTP: Guanosine triphosphate
HRAS: Harvey rat sarcoma viral oncogene homolog
ISSVA: International society for the study of vascular anomalies
KRAS: Kirsten rat sarcoma viral oncogene
LPATH: Likely pathogenic
MAPK: Mitogen-activated protein kinase
MAP2K1: Mitogen-activated protein kinase kinase 1
mtor: Mammalian target of rapamycin
NRAS: Neuroblastoma ras viral oncogene homolog
PATH: Pathogenic
PI3K: Phosphatidylinositol-3-kinase
PIK3C: Phosphatidylinositol-4,5-bisphosphate 3-kinase catalytic subunit alpha
PV: (Likely) Pathogenic variant
RAF1: Raf-1 proto-oncogene, serine/threonine kinase
RASA1: RAS p21 protein activator 1
RIT1: Ras like without CAAX 1
PTEN: Phosphatase and tensin homolog
SOS1: Son of sevenless homolog 1
STREGA: Strengthening the reporting of genetic association studies
TIRM: Turbo-inversion-recovery-magnitude
TSE: Turbo-spin-echo
TWIST: Time-resolved-angiography-with-interleaved-stochastic-trajectories
VUS: Variant of uncertain significance
