# Supplementary material for: Detection rate and mutational landscape in extracranial arteriovenous malformations: a cohort study
Source: BMC Med. 2026 Apr 16;24:248. doi: 10.1186/s12916-026-04874-0 (PMC13085549; doi:10.1186/s12916-026-04874-0)
Supplement: Supplementary file 2 — Additional file 2. Table 2 Mutational spectrum and genotypic characterization of study cohort. [file 12916_2026_4874_MOESM2_ESM.docx]

**Supplemental Table 1. Mutational spectrum and genotypic characterization of study cohort**

| **Pat.No.** | **Gene** | **cDNA change** | **Protein change** | **Source** | **Technology** | **Mutant allele fraction** | **Mutant allele count** | **Total  allele  count** | **ACMG class** | **Causative variant** | **Previously published*** |
| --- | --- | --- | --- | --- | --- | --- | --- | --- | --- | --- | --- |
| 1 | *KRAS* | c.35G>A | p.Gly12Asp | FT | Somatic panel | 8.2% | 104 | 1273 | PATH | definite | yes |
| 2 | *KRAS* | c.183A>C | p.Gln61His | FT | Somatic panel | 6.6% | 135 | 2024 | PATH | definite | yes |
| 3 | *KRAS* | c.35G>A | p.Gly12Asp | FT | Somatic panel | 6.3% | 173 | 2727 | PATH | definite | no |
| 4 | *KRAS* | c.35G>A | p.Gly12Asp | FT | Combined panel | 5.4% | 162 | 2989 | PATH | definite | no |
| 5 | *KRAS* | c.35G>A | p.Gly12Asp | FT | Somatic panel | 7.3% | 343 | 4688 | PATH | definite | no |
| 6 | *KRAS* | c.35G>A | p.Gly12Asp | FT | Somatic panel | 14.2% / 8.6% | 661 / 210 | 4661 / 2456 | PATH | definite | yes |
| 7 | *KRAS* | c.35G>A | p.Gly12Asp | FT | Combined panel | 7.0% | 170 | 2423 | PATH | definite | no |
| 8 | *KRAS* | c.180_182delinsACG | p.Gln61Arg | FT | Somatic panel | 8.1% | 323 | 3993 | LPATH | definite | no |
| 9 | *KRAS* | c.188_229dup | p.Glu63_Glu76dup | FT | Somatic panel | 4.7% | 122 | 2599 | PATH | definite | yes |
| 10 | *KRAS* | c.35G>A | p.Gly12Asp | FFPE | Somatic panel | 4.5% | 76 | 1703 | PATH | definite | yes |
| 11 | *KRAS* | c.35G>C | p.Gly12Ala | FT | Combined panel | 3.3% | 147 | 4406 | LPATH | definite | no |
| 12 | *KRAS* | c.174_175insATGGATATTCTCGACACA | p.Thr58_Ala59insMetAspIleLeuAspThr | FT | Combined panel | 7.1% | 229 | 3218 | LPATH | definite | no |
| 13 | *KRAS* | c.35G>A | p.Gly12Asp | FT | Combined panel | 2.5% | 78 | 3088 | PATH | definite | no |
| 14 | *KRAS* | c.183A>C | p.Gln61His | FT | Somatic panel | 3.7% | 185 | 4999 | PATH | definite | no |
| 15 | *KRAS* | c.35G>A | p.Gly12Asp | FT | Combined panel | 3.3% | 86 | 2624 | PATH | definite | no |
| 16 | *KRAS* | c.35G>T | p.Gly12Val | FT | Combined panel | 4.9% | 157 | 3216 | PATH | definite | no |
| 17 | *KRAS* | c.183A>C | p.Gln61His | FT | Combined panel | 1.8% | 58 | 3180 | PATH | definite | no |
| 18 | *KRAS* | c.35G>T | p.Gly12Val | FT | Combined panel | 8.0% | 244 | 3020 | LPATH | definite | no |
| 19 | *KRAS* | c.132_218dup | p.Val45_Arg73dup | FT | Combined panel | 2.6% | 19 | 737 | LPATH | definite | no |
| 20 | *KRAS* | c.35G>A | p.Gly12Asp | FT | Somatic panel | 8.7% | 148 | 1698 | PATH | definite | yes |
| 21 | *KRAS* | c.35G>A | p.Gly12Asp | FT | Combined panel | 1.6% | 42 | 2577 | PATH | definite | no |
| 22 | *KRAS* | c.183A>C | p.Gln61His | FT | Somatic panel | 9.4% | 106 | 1130 | PATH | definite | yes |
| 23 | *KRAS* | c.35G>A | p.Gly12Asp | FT | Somatic panel | 1.7% | 76 | 4388 | PATH | definite | no |
| 24 | *KRAS* | c.34G>T | p.Gly12Cys | FT | Germline panel | 8.7% | 47 | 540 | PATH | definite | yes |
| 25 | *MAP2K1* | c.167A>C | p.Gln56Pro | FT | Combined panel | 11.5% | 463 | 4024 | PATH | definite | no |
| 26 | *MAP2K1* | c.171G>C | p.Lys57Asn | FT | Combined panel | 5.4% | 125 | 2332 | PATH | definite | no |
| 27 | *MAP2K1* | c.167A>C | p.Glu56Pro | FT | Somatic panel | 5,6% | 13 | 231 | PATH | definite | no |
| 28 | *MAP2K1* | c.171_185del | p.Gln58_Glu62del | FT | Somatic panel | 0.9% | 15 | 1588 | LPATH | definite | yes |
| 29 | *MAP2K1* | c.167A>C | p.Gln56Pro | FT | Somatic panel | 10.9% | 21 | 192 | PATH | definite | yes |
| 30 | *MAP2K1* | c.171G>T | p.Lys57Asn | FT | Somatic panel | 9.7% | 474 | 4886 | PATH | definite | yes |
| 31 | *MAP2K1* | c.171G>T | p.(Lys57Asn) | FT | Combined panel | 3.5% | 132 | 3476 | PATH | definite | no |
| 32 | *MAP2K1* | c.167A>C | p.Gln56Pro | FT | Somatic panel | 10.7% | 505 | 4722 | PATH | definite | no |
| 33 | *MAP2K1* | c.173_187del | p.Gln58_Glu62del | FT | Combined panel | 6.2% | 381 | 6182 | LPATH | definite | no |
| 34 | *MAP2K1* | c.169_170delinsCC | p.Lys57Pro | FT | Sanger (2x) | 16% / 20% |  |  | LPATH | definite | yes |
| 35 | *MAP2K1* | c.171G>T | p.Lys57Asn | FT | Combined panel | 1.8% | 93 | 5170 | PATH | definite | no |
| 36 | *MAP2K1* | c.171G>T | p.Lys57Asn | FT | Somatic panel | 1.8% | 105 | 5872 | PATH | definite | yes |
| 37 | *MAP2K1* | c.173_187del | p.Gln58_Glu62del | FT | Combined panel | 3.8% | 103 | 2693 | LPATH | definite | no |
| 38 | *MAP2K1* | c.171G>T | p.Lys57Asn | FT | Combined panel | 0.4% | 13 | 3260 | PATH | definite | no |
| 39 | *MAP2K1* | c.167A>C | p.Gln56Pro | FT | Somatic panel | 10.0% | 363 | 3636 | PATH | definite | no |
| 40 | *MAP2K1* | c.171G>C | p.Lys57Asn | FT | Somatic panel | 13.0% | 639 | 4914 | PATH | definite | no |
| 41 | *MAP2K1* | c.171G>C | p.(Lys57Asn) | FT | Combined panel | 10.2% | 311 | 3049 | PATH | definite | no |
| 42 | *MAP2K1* | c.171G>T | p.Lys57Asn | FT | Somatic panel | 4.0% | 215 | 5381 | PATH | definite | no |
| 43 | *MAP2K1* | c.173_187del | p.Gln58_Glu62del | FFPE | Combined panel | 6.0% | 54 | 898 | LPATH | definite | no |
| 44 | *MAP2K1* | c.361T>A | p.Cys121Ser | FFPE, FT | Combined panel | 5.4%; | 197 | 3674 | LPATH | definite | no |
| 45 | *HRAS* | c.172_179delACCGCCGGinsGTCCTGGATGTACT | p.Thr58_Gly60delinsValLeuAspValLeu | FFPE | Somatic panel | 7.0% | 162 | 2331 | LPATH | definite | yes |
| 46 | *HRAS* | c.191_217dup | p.Met72_Arg73insHisSerAlaMetArgAspGlnTyrMet | FT | Somatic panel | 15.0% | 322 | 2146 | LPATH | definite | yes |
| 47 | *HRAS* | c.217_218insCCAGCGCCATGCGGGACCAGTACATGC | p.Met72_Arg73insProSerAlaMetArgAspGlnTyrMet | FT | Combined panel | 10.9% | 733 | 6719 | LPATH | definite | no |
| 48 | *HRAS* | c.217_218ins27 | p.Met72_Arg73insProSerAlaMetArgAspGlnTyrMet | FFPE | Somatic panel | 11.0% | 118 | 1077 | LPATH | definite | yes |
| 49 | *HRAS* | c.199_228dup | p.Met67_Glu76dup | FT | Combined panel | 0.3% | 18 | 6145 | LPATH | definite | no |
| 50 | *HRAS* | c.208_209insGGTGGGAGTACAGCGCCATGCGGGACC | p.Asp69_Gln70insArgTrpGluTyrSerAlaMetArgAsp | FFPE | Combined panel | 9.8% | 378 | 3855 | LPATH | definite | no |
| 51 | *HRAS* | c.215_216insTTCCAGCGCCATGCGGGACCAGTACAT | p.Tyr71_Met72insIleSerSerAlaMetArgAspGlnTyr | FT | Somatic panel | 7.0% | 113 | 1614 | LPATH | definite | yes |
| 52 | *HRAS* | c.208_209insGGTGGTACAGCGCCATGCGGGACC | p.Asp69_Gln70insArgTrpTyrSerAlaMetArgAsp | FT | Combined panel | 10.5% | 646 | 6145 | LPATH | definite | no |
| 53 | *HRAS* | c.172_177delinsGTCCTGGATGTT | p.Thr58_Ala59delinsValLeuAspVal | FT | Combined panel | 6.7% | 37 | 535 | LPATH | definite | yes |
| 54 | *HRAS* | c.207_236dup | p.Gln70_Leu79dup | FT | Combined panel | 8.0% | 423 | 5295 | PATH | definite | no |
| 55 | *BRAF* | c.1799T>A | p.Val600Glu | FT | Somatic panel | 9.3% | 323 | 3483 | PATH | definite | no |
| 56 | *BRAF* | c.1799T>A | p.Val600Glu | FT | Sanger / dPCR | 23% / 15,3% | NA | NA | PATH | definite | no |
| 57 | *BRAF* | c.1799T>A | p.Val600Glu | FT | Somatic panel | 5.7% | 94 | 1656 | PATH | definite | yes |
| 58 | *BRAF* | c.1517+2_1517+3insTACTCAGGT | p.Arg506_Lys707insLeuLeuArg | FT | Somatic panel | 23% | 631 | 2744 | LPATH | definite | no |
| 59 | *BRAF* | c.1799T>A | p.Val600Glu | FT | Somatic panel | 2.8% | 343 | 12296 | PATH | definite | yes |
| 60 | *BRAF* | c.1799T>A | p.Val600Glu | FT | Combined panel | 4.6% | 143 | 3082 | PATH | definite | no |
| 61 | *BRAF* | c.1799T>A | p.Val600Glu | FT | Somatic panel | 4.0% | 71 | 1754 | PATH | definite | yes |
| 62 | *BRAF* | c.1799T>A | p.Val600Glu | FT | Somatic panel | 10.4% | 191 | 1828 | PATH | definite | yes |
| 63 | *BRAF* | c.1799T>A | p.Val600Glu | FT | Combined panel | 7.2% | 352 | 4887 | PATH | definite | no |
| 64 | *RASA 1 germline* | c.2603+1G>A | p.? | FT, EDTA blood | Germline panel | het | - | - | PATH | definite | no |
| 65 | *RASA 1 germline* | c.2977del | p.Arg993Valfs*3 | EDTA blood | Germline panel | het | - | - | LPATH | definite | no |
| 66 | *RASA 1 germline* | c.2450_2451del | p.Ser817Tyrfs*12 | EDTA blood | Germline panel | het | - | - | LPATH | definite | no |
| 67 | *RASA 1 germline* | c.2035C>T | p.Arg679* | Leukocyte DNA | Sanger RASA1 | het | - | - | PATH | definite | no |
| 68 | *RASA 1 germline* | c.2254delC | p.Leu752Trpfs*6 | EDTA blood | Germline panel | het | - | - | LPATH | definite | no |
| 69 | *RASA 1 germline* | c.3028C>T | p.Arg1010* | EDTA blood | Germline panel | het | - | - | PATH | definite | no |
| 70 | *RASA 1 germline* | c.2603+2dup | p.? | EDTA blood | Sanger | het | - | - | LPATH | definite | no |
| 71 | *RASA 1 germline* | c.1466G>A | p.Arg489His | FT | Combined panel | het | - | - | VUS | candidate | no |
| 72 | *RASA 1 somatic* | c.2131C>T; c.337dup | p.Arg711Ter; p.Asp113Glyfs*14 | FT | Combined panel | 6.1% / 5,8% | 226 / 277 | 3714 / 4818 | PATH / LPATH | definite | no |
| 73 | *RASA 1 somatic* | c.2494_2503del; c.1148del | p.Pro832*; p.Pro383Leufs*28 | FT | Combined panel | 7.4% / 5,0% | 43 / 72 | 582 / 1443 | LPATH / LPATH | definite | no |
| 74 | *PTEN germline* | c.987_990del | p.Asn329Lysfs*14 | FT | Germline panel | het | - | - | PATH | definite | no |
| 75 | *PTEN germline* | c.675T>A | p.Tyr225* | FT | Combined panel | het | - | - | PATH | definite | no |
| 76 | *PTEN germline* | c.955_958del | p.Thr319* | EDTA blood | Sanger | het | - | - | LPATH | definite | no |
| 77 | *PTEN germline* | c.370T>C | p.Cys124Arg | EDTA blood | Germline panel | het | - | - | PATH | definite | no |
| 78 | *PTEN germline* | c.328C>T | p.Gln110* | EDTA blood | Sanger | het | - | - | PATH | definite | no |
| 79 | *PTEN germline* | c.741dup | p.Pro248Thrfs*5 | FT | Sanger, Combined panel | het | - | - | PATH | definite | no |
| 80 | *PTEN somatic* | c.697C>T | p.Arg233* | FT | Combined panel | 21.3% | 582 | 2737 | PATH | definite | no |
| 81 | *PTEN somatic* | c.403A>G | p.Ile135Val | FFPE | Somatic panel | 7.3% | NA | NA | PATH | definite | no |
| 82 | *PTEN somatic* | c.469del | p.Glu157Lysfs*2 | FT | Combined panel | 18.0% | 212 | 1177 | LPATH | definite | no |
| 83 | *PIK3CA* | c.1636C>A | p.Gln546Lys | FT | Somatic panel | 3.0% | 4 | 134 | VUS | candidate | no |
| 84 | *PIK3CA* | c.1633G>A | p.Glu545Lys | FFPE | Somatic panel | 11.2% | 311 | 2769 | PATH | definite | no |
| 85 | *PIK3CA* | c.1633G>A | p.Glu545Lys | FT | Somatic panel | 10.9% | 284 | 2595 | PATH | definite | no |
| 86 | *PIK3CA* | c.1258T>C | p.Cys420Arg | FT | Somatic panel | 3.8% | 106 | 2769 | LPATH | definite | no |
| 87 | *EBH4 germline* | c.2608_2615del | p.Val870Argfs*73 | FT | Combined panel | het | - | - | PATH | definite | no |
| 88 | *EBH4 germline* | c.1546G>A | p.Gly561Arg | FT | Germline panel, Somatic panel | het | - | - | VUS | candidate | no |
| 89 | *EBH4 germline* | c.2173G>A | p.Ala725Thr | EDTA blood | Sanger | het | - | - | VUS | candidate | no |
| 90 | *EBH4 germline* | c.2173G>A | p.Ala725Thr | EDTA blood | Germline panel | het | - | - | VUS | candidate | no |
| 91 | *SOS1* | c.1462_1466delinsCTTTTCTTAAAGAAACTTTTCTTAAAGAAAAGTTT | p.Tyr488_Arg489delinsLeuPheLeuLysLysLeuPheLeuLysLysSerPhe | FT | Combined panel | 1.7% | 45 | 2677 | LPATH | definite | no |
| 92 | *SOS1* | c.1462_1467delinsAAGTTTTTTATGCGAAAGTTTTTTATGCGAAAAGTTTTTAAA | p.Tyr488_Arg489delinsLysPhePheMetArgLysPhePheMetArgLysValPheLys | FFPE | Combined panel | 5.2% | 41 | 791 | LPATH | definite | no |
| 93 | *SOS1* | c.1289_1300delinsGACAGACATTGAAAAAAAAAAAAA | p.Asp430_Gly434delinsGlyGlnThrLeuLysLysLysLysArg | FT | Combined panel | 4.9% | 235 | 4790 | LPATH | definite | no |
| 94 | *GNAQ* | c.627A>T | p.Gln209His | FT | Somatic panel | 9.5% | 453 | 4767 | PATH | definite | no |
| 95 | *GNAQ* | c.548G>A | p.Arg183Gln | FT | Somatic panel | 7.1% | 419 | 5939 | PATH | definite | no |
| 96 | *RIT1* | c.246_248delinsCCCTCT | p.Thr83delinsProLeu | FT | Somatic panel | 3.3% | 119 | 3620 | LPATH | definite | no |
| 97 | *RAF1* | c.1171A>T | p.Arg391Trp | FT | Combined panel | 7.0% | 251 | 3081 | VUS | candidate | no |
| 98 | *GNA14* | c.614A>T | p.Gln205Leu | FT | Somatic panel | 15.5% | 441 | 2842 | PATH | definite | no |

aa=amino acid. ACMG=american college of medical genetics and genomics. cDNA=coding deoxyribonucleic acid. dPCR=digital polymerase chain reaction. EDTA=ethylenediaminetetraacetic acid. FFPE=formalin-fixed paraffine-embedded tissue. FT=fresh tissue. het=het. LPATH=likely pathogenic. No.=number. PATH=pathogenic. UMI=unique molecular identifiers.
