# Supplementary material for: Detection rate and mutational landscape in extracranial arteriovenous malformations: a cohort study
Source: BMC Med. 2026 Apr 16;24:248. doi: 10.1186/s12916-026-04874-0 (PMC13085549; doi:10.1186/s12916-026-04874-0)
Supplement: Supplementary file 3 — Additional file 3. Details about DNA extraction, variant screening, and genetic analysis. [file 12916_2026_4874_MOESM3_ESM.docx]

**Additional file 2**

*DNA extraction*

Genomic DNA was extracted from native tissue samples or deparaffinated FFPE samples using the QIAamp DNA Mini Kit (Qiagen, Hilden, Germany) following the manufacturer´s instructions. DNA extraction from venous blood samples was performed according to standard protocols on an QIAcube connect MDx instrument (Qiagen, Hilden, Germany). DNA concentration was measured using a Qubit 4 fluorometer (Invitrogen, Carlsbad, CA, USA) and adjusted according to the requirements of subsequent experiments.

*Variant screening*

A minority of individuals underwent only germline testing of leukocyte DNA samples via targeted Sanger sequencing of *RASA1*/*EPHB4* and *PTEN* due to a strong clinical suspicion of CM-AVM or PTEN hamartoma tumor syndrome (PTHS), respectively (Supplemental Table 1). To this end, exons and flanking intronic regions were amplified by PCR, and bidirectional Sanger sequencing was performed using Big Dye Terminator Cycle Sequencing Kit on a 3500xl Genetic Analyzer (Applied Biosystems, Foster City, CA).

In two tissue samples the causative mosaic PVs with an estimated VAF of 15-20% were detected by Sanger sequencing of MAP2K1 and BRAF hotspot exons and confirmed by repeated testing and digital PCR (dPCR), respectively (Supplemental Table 1: Pat. No. 34 and 56).

The majority of samples were investigated via massive parallel sequencing of targeted multi-gene panels using Illumina short-read technology (Illumina, San Diego, California, USA). Enrichment methods and testing strategies were evolving over time. Initially, separate multigene panels with complementary gene content were used for somatic and germline testing (“Somatic panel” and “Germline panel”), either sequentially or simultaneously, depending on the clinical context. A read depth of 100-300x was targeted for the germline panels. The panels for screening for somatic variants, on the other hand, were sequenced at ultra-high depth with the additional use of UMIs (unique molecular identifiers). A read depth of at least 2000 consensus reads (after demultiplexing) was envisaged in order to achieve a mosaic detection sensitivity of at least 0.5% VAF. These values could not always be achieved with FFPE material. Subsequently, genes with typically germline variants and those with typically somatic variants were merged into one multigene panel using UMIs and ultra-high-throughput sequencing (“Combined panel”). Different target enrichment kits were used during the course of this project, including Illumina TruSeq Custom Amplicon Panel (TSCA); Agilent SureSelect XT HS2 DNA Custom Panel with UMIs (random 3bp duplex) (Agilent Technologies, Santa Clara, CA, USA); Twist EF Custom Library Prep 2.0 of a Twist Custom Panel with Twist UMI Adapter (fixed 5bp or 6bp duplex) (Twist Bioscience, South San Francisco, CA, USA). The target sequence comprised a panel of genes / gene hotspots that are known to be involved in vascular malformations or regional overgrowth.

Library preparation was performed according to the respective manufacturer’s instructions. Indexed sample libraries were equimolarly pooled for final multiplexed sequencing. Sequencing was carried out by paired-end sequencing with 2x151 bp reads on a MiSeq, NextSeq 550 or NovaSeq X Plus system (Illumina, San Diego, California, USA), respectively, to achieve for each sample the desired reading depth, respectively.

If samples tested negative using an older method, retesting with the latest version of the Combined panel was attempted. If the result remained negative, the analysis was designated as “unrestricted”. If the Combined panel could not be used due to insufficient sample material, the analysis was designated as “restricted”. This also applies to cases in which only leukocyte DNA but no lesional tissue sample was available, or in cases with only FFPE material of poor DNA quality, where the analysis remained of limited quality.

*Genomic data analyses*

Raw data (bcl-format, binary base call sequence files) were uploaded to the varvis® software package (Limbus Medical Technologies GmbH, Rostock, Germany) and processed (demultiplexing, read alignment, error correction) using the GRCh37 reference genome and the varvis® bioinformatics pipeline in its latest version available at the time, most recently version 2.4.1. The manufacturer's standard settings were applied. For UMI analysis (duplex sequencing data processing), duplex barcode sequences were extracted from the read sequence according to the manufacturer's user manual (Agilent SureSelect XT HS2 DNA Kits Protocol or Twist EF Library Preparation Kit, respectively) and reads were aligned to the reference genome. A minimum of two reads were required to define a strand-specific consensus read. Strand-specific consensus reads were then combined to create a final consensus read. Variant consensus reads were called down to a minimum variant allele frequency (VAF) of 0.5% with two aberrant consensus reads minimum, thereby reaching a detection threshold for mosaic variants of at least 0.5% for most samples. The target regions typically had a mean sequencing depth of >2,000x after demultiplexing, except for DNA samples from FFPE tissue which yielded variable but usually lower coverage.
